# Flipons and the origin of the genetic code

**DOI:** 10.1098/rsbl.2024.0635

**Published:** 2025-01-22

**Authors:** Alan Herbert

**Affiliations:** ^1^ Discovery, InsideOutBio, Charlestown, MA, USA

**Keywords:** flipons, evolution, genetic code, DNA, RNA, tinkers

## Abstract

This paper is focused on the origins of the contemporary genetic code. A novel explanation is proposed for how the mapping of nucleotides in DNA to amino acids in proteins arose that derives from repeat nucleotide sequences able to form alternative nucleic acid structures (ANS), such as the unusual left-handed Z-DNA, triplex, G-quadruplex and I-motif conformations. The scheme identifies sequence-specific contacts that map ANS repeats to dipeptide polymers (DPS). The stereochemistry required naturally evolves into a non-overlapping, triplet code for mapping nucleotides to amino acids. The ANS/DPS complexes form a simple, genetically transmitted, self-templating, autonomously replicating collection of ‘tinkers’ for Nature to evolve. Tinkers have agency and promote their own synthesis by forming catalytic scaffolds with metals, further enhancing their capabilities. Initial support for the model is provided by computational models built with AlphaFold3. The predictions made are properly falsifiable with the currently available methodology.

## 1. Introduction

Transmission of information from one generation to the next underlies the origins of life and evolves through Nature’s incessant tinkering with whatever is immediately available [1]. The problem of life’s origins dates back to the work of Urey and Miller, who demonstrated that many of the necessary components can form under prebiotic conditions [2]. Subsequent additions to the Urey and Miller brew of ingredients, such as hydrogen sulfide, later were shown to greatly expand the precursor pool [3,4]. Starting with Darwin [5], many schemes were advanced to explain how such a ‘prebiotic soup’ could have bootstrapped life’s more complex chemistries [6–8]. Many of the debates focused on whether the first informational molecules were crafted from inorganic materials, from proteins or peptides or were actually dependent on either DNA, RNA or another novel chemistry, or derived from extra-terrestrial sources [9]. However, none of these proposed models in their current form fully account for the origins of the contemporary genetic code (**cgc**) (more correctly called a cipher) that maps triplet codons to amino acids [10–13].

Francis Crick in his ‘frozen code’ paper identified three steps in the construction of the **cgc**, tracing its development from primitive to intermediate to the final form [10]. We now know the final form of the **cgc** to be universal, with some exceptions due to differences in stop codon usage, reflecting the competition between hosts and pathogens [14]. Advances in understanding the intermediate stages became possible once more phylogenetically diverse DNA and protein sequences were gathered. A highly significant development was the proposal by Rodin and Ohno that the two families of amino acid tRNA synthetases (aaRS) used to link amino acids to tRNA arose from a single gene. Even more startling was the finding that each strand of the DNA for that single gene generates a separate class of aaRS [15], with 10 amino acids charged by each aaRS family (table 1). Interestingly, the two aaRS classes have different preferences for the middle letter of the codon: class I enzymes preferentially acylate the 2′OH and prefer a uridine base at the second codon position (with the exception of phenylalanine (F)), while the class II enzymes preferentially acylate the 3′OH and recognize cytosine in position 2 [16]. Further, 9 of 10 anticodons for the main basic amino acids, arginine (R), histidine (H) and lysine (K), have a complementary partner, which is recognized by aaRS from the opposite class (the exception is K and F) (table 1) [15]. The closely related aaRS necessitates a secondary, protein-based discriminatory mechanism to ensure that each tRNA is charged with the correct amino acid [17]. The detail of how exactly each family evolved remains unresolved [13]. Possibly, a gene duplication gave rise to an inverted repeat that underwent N-terminal fusion with its parent, generating class I aaRS from class II, with one class II sequence block conserved and another class I sequence encoded by a complementary strand [13,15].

**Table 1.** All class I tRNAs and the class II phenylalanine tRNA are charged on the 2′OH group. The other class II tRNAs are charged on the 3′OH group. The single-letter IUPAC nomenclature for each amino acid is given within parentheses.

class I	class II
tyrosine (Y)	phenylalanine (F)
isoleucine (I)	serine (S)
methionine (M)	proline (P)
valine (V)	threonine (T)
cysteine (C)	alanine (A)
glutamine (Q)	asparagine (N)
arginine (R)	lysine (K)
glutamic acid (E)	aspartic acid (D)
tryptophan (W)	histidine (H)
leucine (L)	glycine (G)

Understanding Crick’s primary step in the evolution of the **cgc** has proven more difficult. Many of the proposed schemes are unbounded, with far too many possible permutations to explore experimentally. Recent computational approaches can help by excluding energetically unfavourable scenarios and by generating experimentally testable hypotheses. These approaches include those implemented in the AlphaFold3 algorithm and by other open-source alternatives [18,19]. The trade of computational time for experimental time helps deploy limited wet lab resources more efficiently.

Simple scenarios can easily be modelled *in silico*, starting with the assumption that the initial code was based on a limited number of repetitive sequence motifs. Not only is the search space much smaller, but the self-templating and self-replicating entities will also have lower algorithmic complexity. For example, self-complementary nucleic acid repeats can template their own Watson–Crick replication [20], with repeated cycles of dissociation and synthesis leading to amplification of the nucleotide sequence. Conceptually, the scheme involves simple condensing agents like carbodiimides to grow the polymers combined with temperature fluctuations to melt the duplexes produced [21]. Others have proposed a variety of non-enzymatic chemistries that enable polymeric peptides to template their own synthesis [22,23], noting that under prebiotic conditions, peptides are more stable than RNA [23]. Xenobiologics have also received attention as monomers can condense to form self-replicating oligomeric aggregates following the exposure of reactive groups by ring opening [24].

One suggested scenario for generating the genetic cipher involves a direct stereochemical match between nucleotides and amino acids. The approach originates with the diamond code of George Gamow [25,26]. The strongest support for the latest versions of this hypothesis is provided by the statistical match between ribosomal codons and the amino acids they specify [27]. An even earlier model proposed by Astbury noted a match between the nucleic acid repeat spacing and that of the β-fold of extended fibres, suggesting that this interaction was of significance. At the time, neither the structure of DNA nor that of β sheets was known [28]. This intuition was later confirmed when more detailed structures became available for modelling. A β-ribbon was found to dock into the minor groove of B-DNA [29]. In this scheme, the authors proposed that there was sufficient room in the B-DNA helix to dock all the amino acid side chains, except for proline and tryptophan. The model derived from another that was based on dsRNA, where a right-handed, antiparallel β-polypeptide-ribbon was proposed to dock within the deep major groove of the RNA helix. The authors commented that in this scheme, it was not evident how the nucleotide sequence mapped to the amino acid coding. Nor was it clear how the β-sheet peptide residues pointing away from the dsRNA helix were mapped. Importantly though, the authors noted that the RNA and protein polymers might act as ‘primordial polymerases’ to drive the synthesis of each other [30].

Here, I focus on a different stereochemical scheme by which nucleotide and peptide polymers promote the formation of each other. The interactions described lead directly to the **cgc**. The models are based on alternative nucleic acid structures (ANS) that can form under the non-physiological conditions present in the prebiotic soup, ranging from high salt to low pH conditions [31,32]. The ANS folds are encoded by sequences, called flipons, that most simply consist of low-complexity nucleotide repeats that can adopt various helical conformations under different conditions [33]. Examples of flipons include left-handed Z-DNA that is formed by alternating purine–pyrimidine repeats such as d(CG)_n_ [34], triplexes formed by homopolymer repeats based on d(ATA), d(TAT) and d(GCG) triplets [35], G-quadruplexes (GQ) based on guanine repeats that form tetrads [36] and four-stranded I-motifs [37] formed by the intercalation of protonated cytosine base pairs.

## 2. Motivation

**Figure 1.**
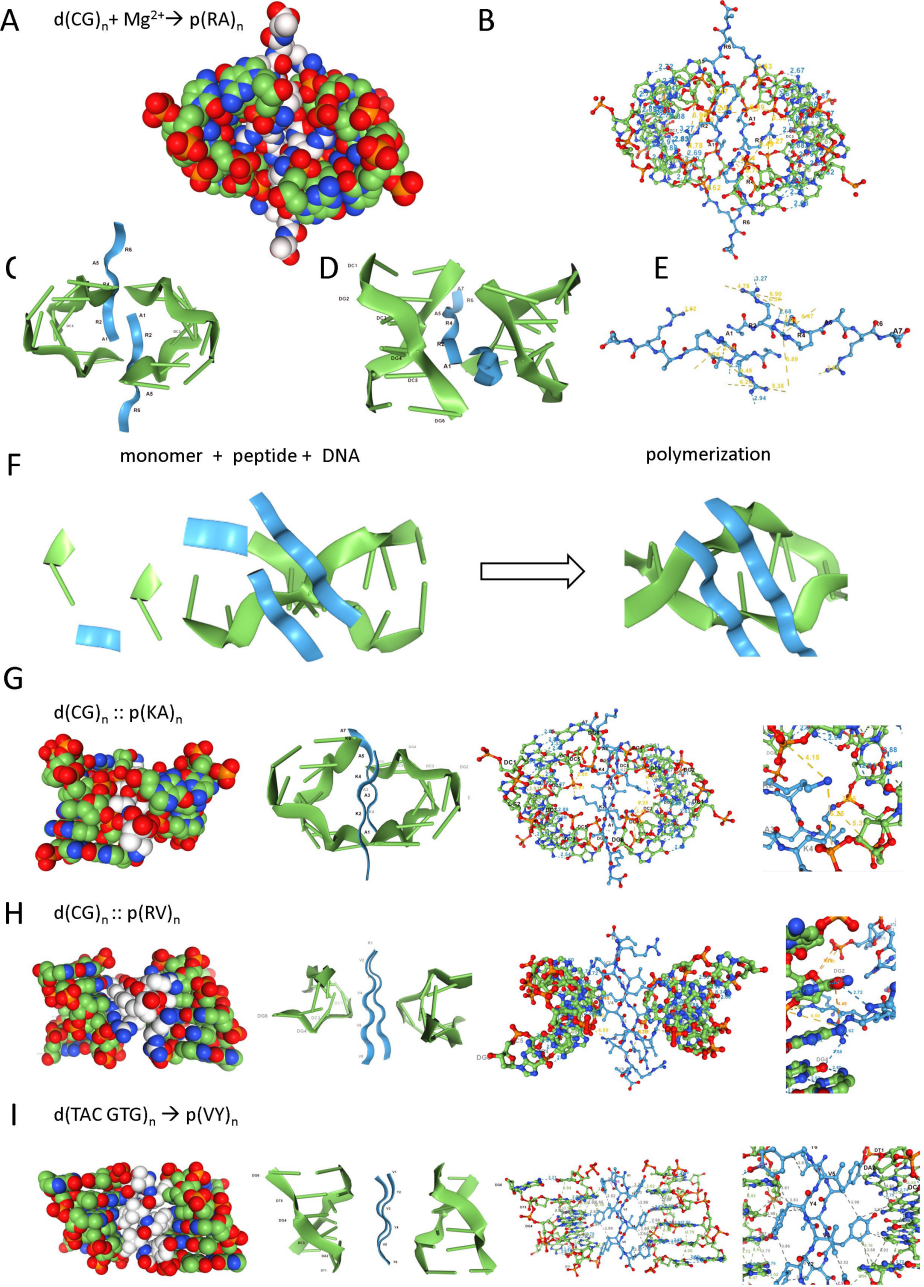
The d(CG)_n_ repeat favours the formation of left-handed Z-DNA and templates the assembly of the arginine–alanine p(RA)_n_ peptide that the sequence encodes. Here, as with the subsequent figures, many different representations of the interactions between flipon structures and dipeptide polymers are presented to emphasize the key features involved. PDB files are given as electronic supplementary material for readers to explore these interactions in more depth. (*a*) A Z-DNA dimer shown by a space-filling model is formed by a p(RA)_n_ β-sheet that binds across the Z-DNA groove. (*b*) The bonding scheme of the dipeptide to Z-DNA and to itself. (*c,d*) Different representations of the interactions between p(RA)_4_ and d(CG)_6_. (*e*) Peptide-mediated bonding scheme, with yellow indicating ionic interactions and grey showing backbone hydrogen bonds that hold the dimer together. The bond lengths in this and subsequent figures are in Å. The salt bridge distances shown are between Nε of arginine and the DNA phosphate O1 atom, setting the NGL Viewer 2.0.0-dev.37 parameter refineSaltBridges = T, while the bridge formed by the phosphate O2 atom is less than 4 Å. (*f*) A model where the DNA double helix and the β-sheet peptide dimer act to template the synthesis of nucleotide and protein polymers. (*g*) The p(KA)_n_ dipeptide docks across the groove of d(CG)_n_ but does not make base-specific contacts. (*h*) The p(RV)_n_ dipeptide binds to the convex surface of d(CGCGTG)_n_ polymer that encodes its amino acids. (*i*) p(VY)_n_ also binds to the convex surface of the Z-DNA sequence d(TACGTG)_n_ through CH–π hydrogen bonds to their C8 position. The valine residues orientate the tyrosine residues.

The work started serendipitously using AlphaFold3 [18] to evaluate a set of peptides reported to interact *in vitro* with the left-handed Z-DNA helix. These peptides have alternating lysine or arginine residues that induce the flip of right-handed B-DNA polymers to left-handed Z-DNA [38] (figure 1). Modelling of different variations of the repeat revealed that a β-sheet formed by an arginine–alanine repeat p(RA)_n_ (p indicates a peptide) provided optimal docking to Z-DNA. In the structures, the p(RA)_n_ dimer binds across the deep groove of the left-handed helix, with arginines inserted into the gap and making base-specific contacts with the O_2_ of cytosine (figure 1*a*–*e*).

Intriguingly, the d(CG)_n_ repeat not only has the highest propensity of any sequence to form Z-DNA but also encodes the RA dipeptide that promotes Z-DNA formation [39,40]. There is no *a priori* reason why the CGC triplet should encode arginine, nor why the GCG triplet should encode alanine. However, the association between a nucleotide repeat and its peptide product easily explains the origins of the **cgc** and its properties. Importantly, the physical nature of the interaction shown in figure 1 ensures that any **cgc** that maps d(CG)_n_ to p(RA)_n_ is non-overlapping. Further, if a repeat is to specify a dipeptide, the number of nucleotides in a repeat must be odd as an even-numbered repeat in a non-overlapping code would always map to a single amino acid, regardless of its length. Indeed, it was this relationship that originally provided the key to deciphering the **cgc** [41–44]. The stereochemistry of mapping d(CG)_n_ to p(RA)_n_ then yields a triplet genetic code through a three-dimensional alignment that projects to the known one-dimensional linear codec.

The interaction between the dipeptide β-sheet of p(RA)_n_ and Z-DNA is stabilized by properties unique to each polymer. The alignment of DNA bases is favoured by stacking interactions and interstrand hydrogen bonding, while the peptide chains are zipped together into a β-sheet stabilized by both ionic and hydrogen bonds. The dimer-on-dimer DNA–peptide interactions provide a shared interface that potentially templates the sequence-specific addition of monomers to each polymer (figure 1*f*). The amino acid and nucleotide precursors are retained in place for longer than would occur otherwise, allowing more time for the condensation reactions to complete. As the protein–DNA interface is relatively small compared to the length of each polymer chain, stochastic processes can easily separate the two products to increase the turnover rate. In this scheme, the DNA facilitates the reactions necessary to extend the β-sheet and the peptide promotes elongation of the DNA strands by correctly orientating the backbone residues.

The interaction is specific for p(RA)_n_, as p(KA)_n_ and p(HA)_n_ that also contain positively charged side chains do not yield a similar complex: p(KA)_n_ binds across the groove and inserts lysine into the gap, but without creating base-specific contacts (figure 1*g*), while dimer formation between Z-DNA and p(HA)_n_ is not observed. The p(RV)_n_ dipeptide binds across the minor groove of d(CG)_n_, but valine likely prevents arginine from reaching deep enough into the space to make base-specific contacts. Instead, p(RV)_n_ bonds specifically to the exocyclic groups exposed on the convex surface of Z-DNA formed by d(GC)_n_ (figure 1*h*). This dipeptide is coded by d(CGC GTG), with the interaction illustrating that the stereospecific code can tolerate sequence variations. Interestingly, the larger p(VY)_n_ β-sheet also docks to the convex surface of Z-DNA formed by the d(TAC GTG)_n_ coding sequence, mostly through CH–π hydrogen bonds to the C8 position of adenosine (figure 1*i*).

**Figure 2.**
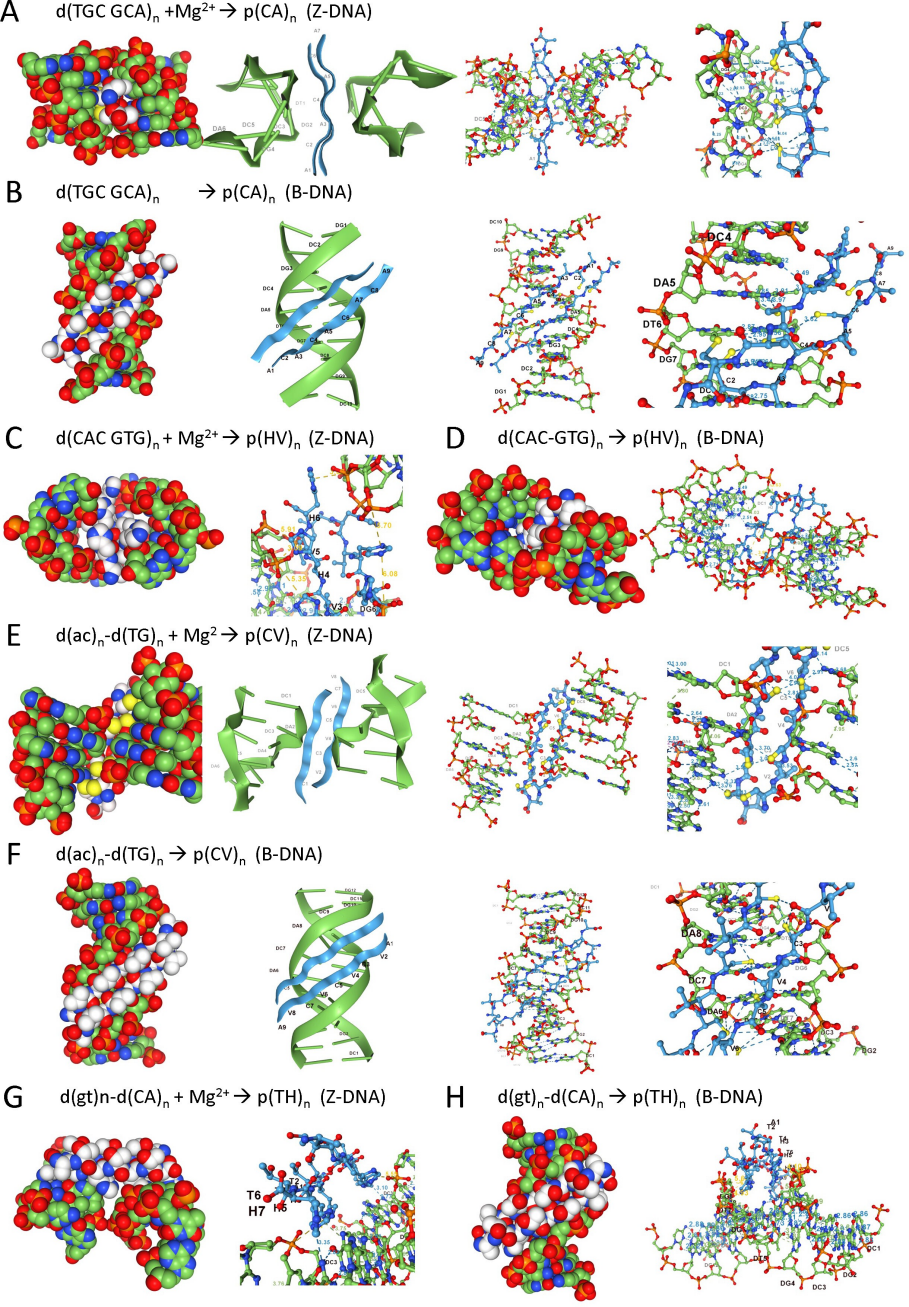
Binding of peptides to the DNA of the sequences encoding them, and the dependence on flipon conformation. (*a*) Space-filling model of the p(CA)_n_ (white carbons) bound to a sequence d(TGC CGA)_n_ (green carbons) that codes for this dipeptide. The interaction in the presence of magnesium is with the convex surface of Z-DNA and involves sequence-specific interactions with N4 of cytosine and O6 of guanosine. The contact details are shown using ribbon and ball-and-stick models. (*b*) In the absence of magnesium, the dipeptide makes sequence-specific contacts in the minor groove of the B-DNA conformer. (*c*) The d(CAC GTG)_n_ sequence codes for p(HC)_n_ that binds to the phosphate backbone across the Z-DNA groove without sequence-specific contacts. (*d*) The same sequence fills the gap between the major groove of B-DNA of one helix and the minor groove surface of another B-DNA duplex. The dipeptide contacts in the major groove are base-specific. (*e*) The d(TG)_n_ repeat encodes the p(CV)_n_ dipeptide and binds to the convex face of Z-DNA in the presence of magnesium through base-specific contacts with adenosine and guanosine, as well as with the phosphate backbone. It also engages the minor groove of B-DNA, making contacts with the N3 of adenosine. (*g*) In the presence of magnesium, the p(TH)_n_ dipeptide docks to N4 of cytosine exposed on the convex surface of the d(CA)_n_ repeat that encodes these amino acids. (*h*) The dipeptide forms an arch over the minor groove of the same sequence in the B-DNA conformation that is bound in the absence of magnesium.

A different complementary codon pair d(TGC GCA)_n_ encodes the p(CA)_n_ dipeptide, which binds in a sequence-specific manner to both Z- and B-DNA flipon conformations. When modelling is performed in the presence of Mg^2+^, the dipeptide docks on the convex face of the Z-DNA helix, binding to the exposed adenosine N6 exocyclic groups (figure 2*a*). When Mg^2+^ is omitted, the dipeptide reaches into the minor groove of B-DNA, contacting both adenosine and thymidine through base-specific interactions (figure 2*b*).

Another self-complementary codon pair d(CAC GTG)_n_ encodes the dipeptide p(HV)_n_. By either excluding or including Mg^2+^ in the model, it was possible to make a direct comparison of the dipeptide interaction with B- and Z-DNA flipon conformation. With Z-DNA, the dipeptide binds across the groove, but is too large to make sequence-specific contacts within the interior space (figure 2*c*). With B-DNA, the dipeptide fills the gap between the major groove of one helix and the minor groove surface of the other. The dipeptide makes base-specific hydrogen bonds to N4 of cytosine of the first helix (figure 2*d*) and backbone contacts with the second.

Many other sequence repeats are also capable of forming Z-DNA, including the d(TG)_n_–d(AC)_n_ DNA duplex [45]. Each strand of d(TG)_n_–d(AC)_n_ encodes a distinct dipeptide that differs in its interactions with B- and Z-flipon conformers. The p(CV)_n_ dipeptide specified by the d(TG)_n_ strand binds over the Z-DNA groove but makes only structure-specific contacts (figure 2*e*). In contrast, with B-DNA, the dipeptide docks in a sequence-specific manner within the minor groove the d(TG)_n_ helix (figure 2*f*). The base contacts are mediated by the cysteines present on one face of the p(CV)_n_ β-sheet, while the valines on the other face of the sheet point away from the DNA axis. The d(AC)_n_ strand of the d(TG)_n_–d(AC)_n_ DNA duplex encodes the much larger p(TH)_n_ dipeptide, with sequence-specific contacts formed over the convex surface of Z-DNA. The β-sheet can bridge two Z-DNA helices (figure 2*g*), but does not make any sequence-specific contacts with B-DNA. Unable to reach into the minor groove, the dipeptide instead forms an outward-facing arch anchored by the phosphate backbone (figure 2*h*). In contrast, the p(CT)_n_ peptide encoded by d(TGTACA)_n_ was unable to bind Z-DNA, but instead formed a lattice-work of sequence-specific contacts in the minor groove of the B-DNA conformer (electronic supplementary material).

The different nucleotide repeats map to specific amino acids in a structure-dependent manner that is dependent upon the flipon conformation. This arrangement creates the necessary diversity to generate a primitive genetic code (**pgc**), where these complexes potentially promote their own self-replication. However, the low efficiency of peptide condensation compared to that mediated by RNA and protein enzymes has been recently noted [17]. For these complexes to replicate, it seems that some form of catalysis was required to enhance their synthesis.

**Figure 3.**
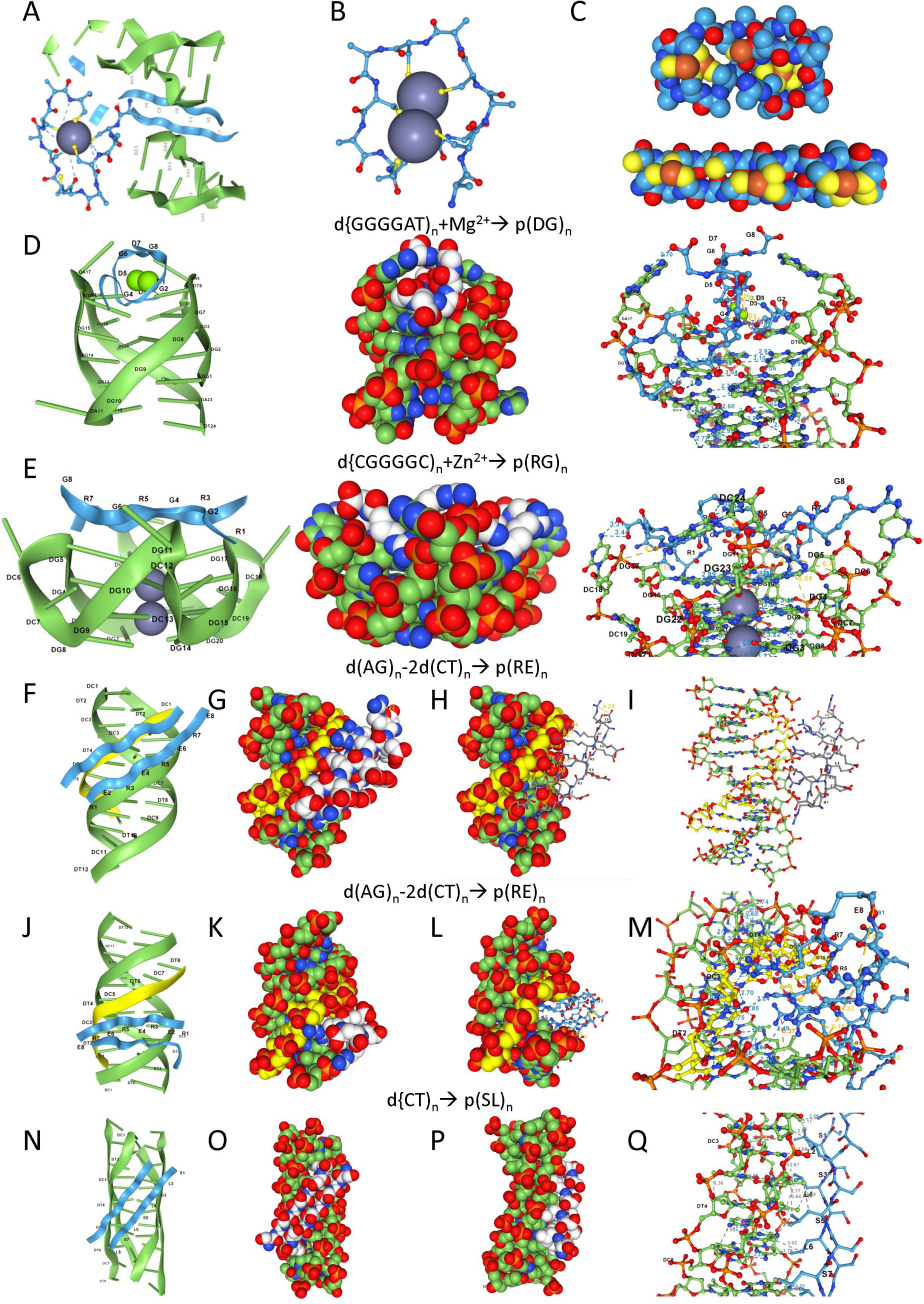
Metal binding by dipeptides. (*a*) Metals bound to dipeptides may catalyse the polymerization of tinkers, enhancing their replication. (*b,c*) Examples of zinc and ferrous clusters that are scaffolded by cysteine-containing dipeptide repeats with varying lengths and folds. (*d*) Coordination of magnesium enhances the interaction of p(DG)_n_ with the GQ formed by the d(GGG GAT)_n_ sequence encoding the repeat. (*e*) Docking in the presence of Zn^2+^ of a p(RG)_4_ to a GQ composed of d(CGGGGC)_4_. (*f–i*) A polypyrimidine antiparallel triplex formed by d(AG-(TC).(TC))_n_ is bound by p(RE)_n_ encoded by d(AG)_n_ . In this model, the peptide tracks with the curvature of the inserted d(TC)_n_ third strand. (*j–m*) An alternative model with the insertion of the p(RE)_n_ dipeptide into the open major groove of the triplex. This interaction generates many base-specific contacts. (*n–q*) Hydrophobic contacts between p(SL)_n_ and the d(CT)_n_ coding sequences that folds to form an I-motif.

An interesting feature of the dipeptide polymers discussed so far is their ability to cluster metals through the cysteine and histidine repeats in order to create catalytic centres (figure 3*a*–*c*). Like true tinkers, these complexes can work productively with metals. The model in figure 3*a* displays how the binding of a metal by a dipeptide might catalytically promote the addition of monomers to extend the tinker polymers. Both zinc (figure 3*b*) and iron (figure 3*c*) can form metal–sulfur clusters. Intriguingly, recent work shows that both cysteine and histidine were synthesized in the first prebiotic soups, with both amino acids proposed to play an important role in the early elaboration of biopolymers [3,46]. Further, the analysis of ancient proteins supports the presence of cysteine and methionine as components of the **pgc**, appearing much earlier in the biosphere than previously thought [4].

The p(DG)_n_ dipeptide that coordinates Mg^2+^ is also likely an early feature of the **pgc** [47]. Importantly, the p(DGD) motif is present in a number of DNA polymerases, consistent with a role in the early synthesis of tinkers. The d(GGGGAU)_n_, guanosine-rich codon for this motif forms a four-stranded GQ that tightly docks two p(DG)_n_ strands to the end plate through Mg^2+^-mediated, base-specific contacts with DNA (figure 3*d*). Metals such as Zn^2+^ also promote the docking of p(RG)_n_ repeats to the GQ formed by its d(CGG-GGC)_n_ coding sequence (figure 3*e*). This is also a well-documented interaction in the contemporary world [48,49].

Other potential examples exist of dipeptides that bind to a GQ formed by their coding sequences. The p(GW)_n_ motif that is present in many RNA-binding proteins, docks to the d(TGGGGT)_n_ through π–π stacking, while p(GK)_n_, p(GN)_n_ and p(GQ)_n_ complexes are stabilized by a network of hydrogen bonds (electronic supplementary material). Some of these interactions involve peptide modifications. For example, a p(PG)_n_ dipeptide containing 4-hydroxyproline (4hP) will dock to the d(GGTCCG)_n_ GQ helix formed by their codons (electronic supplementary material). The 4hP modification is found in many proteins, especially in collagen that evolved before the protostome–deuterostome split at the dawn of metazoa [50,51]. Longer 4hP (and 3hP) peptide chains have a propensity to approximate the amino and carboxyl termini on the GQ scaffold, perhaps also promoting the formation of cyclic peptides that are found in some species [52]. The various interactions with a range of dipeptides also may underlie the diverse functions these structures fulfil in the contemporary cell [53].

Sequence repeats that form triplets represent another type of flipon template specific for the dipeptides that they encode. Notably, the p(RE)_n_ dipeptide engages the polypyrimidine d(AG-(TC).(TC))_n_ triplex formed by its codons (figure 3*f*–*m*). This structure is based on the AGA codon for arginine, rather than the d(CGN) codons derived from the d(CGG) initially used to form GQ, and perhaps accounts for the quirk in the **cgc** where arginine is encoded by two sets of unrelated codons. Modelling with the d(AG-(TC).(TC))_n_ T-flipon gave rise to two different structures: one in which the curve of the β-sheet follows that of the triplex backbone (figure 3*f*–*i*) and the other in which the dipeptide docks in the large triplex groove and makes extensive base-specific interactions (figure 3*j*–*m*). The first structure reflects the orientation of the longer R set by the shorter E amino acid groups, a fold that appears unique to this dipeptide combination. Another interesting variation involves the p(SL)_n_ polymer that binds to an I-motif structure formed by its d(TC)_n_ coding sequence through the hydrophobic leucine face of the β-sheet it forms (electronic supplementary material).

Other schemes based on the use of modified amino acids that dock to ANS are possible. The 4-amino-L modification of phenylalanine (4aF) may act similarly to melamine and stabilize the d(T)n duplex that encodes p(F)_n_ [35,36]. Likewise, modified amino acids like selenomethionine that binds ATG [54] and selenocysteine that binds the TAG stop codon may also engage polymers containing those codons in a sequence-specific manner [55]. Indeed, the replacement of cysteine by selenium would help overcome the extremely weak hydrogen bonding present in the model of p(MC)_n_ bound to the minor groove of d(ATG-TAG)_n_ B-DNA (electronic supplementary material). There are two other stop codons (TAA, TAG) present in the **cgc**. Neither triplet may form stable enough ANS required to promote the synthesis of long amino acid polymers and instead may be prone to generating only di- and tripeptides [44].

## 3. Summary

The mapping proposed here of amino acids to modern-day codons is based on interactions with alternative flipon conformations with dipeptides, some of which coordinate metal ions capable of catalysis. The DNA and protein templates described have the potential to promote the formation of each other and evolve as Nature’s self-replicating ‘tinkers’ (figure 1*f*) [1]. The scheme provides a plausible scenario for the initial mapping of the primitive genetic code envisioned by Crick. One set of these dipeptides is encoded by codons that are self-complementary to each other, and therefore capable of forming duplex DNA structures. As with the Rodin and Ohno scheme, one of these codons is specified by a class I aaRS and the other by a class II aaRS [15]. The interaction of theDNA with the dipeptide encoded depends on both faces of the β-helical strand, either through direct contact or through steric hindrance. This mapping of codons to amino acid sequence thereby gives rise to the **cgc**.

The involvement of only a single DNA and a single unstructured peptide distinguishes the interaction with GQ from those involving Z-DNA. The GQ fold is quite stable relative to Z-DNA and its preformation may lower the entropic cost for using this structure to promote elongation of glycine-containing dipeptides. The variety of GQ folds also increases their potential for biological exploitation [53]. While glycine itself does not specifically bind to GQ, its presence enables the folds necessary for the specific interactions of other amino acids to occur, while precluding the formation of β-sheets that bind to a different DNA conformation. Further, the glycine-rich dipeptides likely promote GQ accumulation by preventing their loss through the degradation of the less stable B-DNA conformer, rather than by directly stimulating their synthesis. The tinkers formed are then able to exploit the ability of GQ to coordinate with a multitude of ionic metals to create catalytic cradles capable of quite complex chemistries [56].

The models described here are just that. Each one requires further experimental validation. That work is possible using synthetic substrates, nucleotide and peptide analogues and the chemistries of the prebiotic world [23]. Initial assessments might test the ability of nucleic and peptide oligomers to catalyse the synthesis of each other, as well as structural studies to examine the interactions at atomic resolution. It might be necessary to examine a range of prebiotic soups that contain metals to optimize outcomes. The harsh conditions under which many ANS are stable may facilitate the formation early in the evolution of large peptide–nucleic acid condensates that increase the efficiency of the reactions required for polymer formation. Interestingly, the primordial ribosome may have translated DNA rather than RNA [57]. Later embellishments, such as tRNA-like adaptors that are engaged by nucleic acid single-stranded overhangs, would boost replication rates by increasing the local concentration of amino acid substrates. The transition to translating RNA templates, which are highly sensitive to metal-induced hydrolysis, likely was favoured by RNA-catalysed peptide bond synthesis. Currently, determining the details of such reaction schemes is beyond the capabilities of current computer simulations, even given the great advances in these approaches that have recently been demonstrated [58].

There may be other possible mappings between ANS and amino acids not described here or discoverable in the current implementation of AlphaFold3. Duplex RNA or some other nucleic acid polymer may also provide a suitable template for the initial mapping of amino acids to codons without directly involving DNA. Junctions or other mismatches that open a nucleic acid helix could also enable base-specific docking of dipeptide polymers or the formation of RNA catalytic moieties, but such processes are not well modelled by the tools used here. Overall, the proposed scheme does not require a DNA, RNA or peptide world to explain life’s origins. Instead, the tinkers described are agents that promote this eventuality. They arise from the simple match between low-complexity nucleotide and simple peptide polymers, using metals to catalyse their initial replication. By spiking the prebiotic soup with copies of themselves, these tinkers quite naturally evolved a non-overlapping, triplet genetic code.

## Data Availability

The models generated by AlphaFold3 [18] are given in Protein Database Format in the electronic supplementary material. Figures were prepared using NGL Viewer [59]. Supplementary material available online [60].
